# Erythema Multiforme as Early Manifestation of COVID-19: A Case Report

**DOI:** 10.3390/pathogens11060654

**Published:** 2022-06-07

**Authors:** Gaspare Palaia, Elena Pernice, Daniele Pergolini, Alessandra Impellizzeri, Guido Migliau, Gianluca Gambarini, Umberto Romeo, Antonella Polimeni

**Affiliations:** Department of Oral and Maxillofacial Sciences, Sapienza University of Rome, Via Caserta 6, 00161 Rome, Italy; gaspare.palaia@uniroma1.it (G.P.); pernice.elena16@gmail.com (E.P.); alessandra.impellizzeri@uniroma1.it (A.I.); guido.migliau@uniroma1.it (G.M.); gianluca.gambarini@uniroma1.it (G.G.); umberto.romeo@uniroma1.it (U.R.); antonella.polimeni@uniroma1.it (A.P.)

**Keywords:** COVID-19 infection, erythema multiforme, oral ulcers, steroid therapy

## Abstract

**Background:** Coronavirus disease 2019 (COVID-19), caused by severe acute respiratory syndrome coronavirus 2 (SARS-CoV-2), is a viral infection involving multi-organ manifestations. The main oral symptoms of COVID-19 associated are taste loss and xerostomia, but literature has reported other oral manifestation, such as oral blisters, ulcers, vesicles and other immunological lesions. This case report showed an Erythema Multiforme (EM) manifesting as oral mucosa lesions in a patient with a late diagnosis of COVID-19 infection. **Case Presentation**: A 30 years-old Caucasian woman was sent to an oral medicine office, in order to manage painful and oral mucosa lesions associated with target symmetrical skin lesions. Oral examination revealed extensive ulcers in the mouth and crusts on the lips. Based on clinical examinations, a diagnosis of Erythema Multiforme major was made and a drug therapy with steroids was administered. Five days after the specialist visit, the patient discovered that she was positive for COVID-19. The complete recovery occurred in 3 weeks. **Conclusion**: Confirming the literature studies, EM is an early disease associated with COVID-19 infection.

## 1. Introduction

The outbreak of coronavirus disease 2019 (COVID-19) caused by severe acute respiratory syndrome coronavirus 2 (SARS-CoV-2) has had an important impact on the approach taken by many medical specialties. This viral infection mostly involves the respiratory system, but it was also found with multi-organ manifestations, including oral mucosa [1].

The biological rationale is that the Angiotensin-Converting Enzyme 2 (ACE2) [1], the main host cell receptor of SARS-CoV-2, is highly expressed on the epithelial cells of the tongue and of the salivary glands, which may suggest a role for oral mucosa in the penetration of the virus into the organism, simultaneously explaining the occurrence of ageusia as one of the first symptoms of COVID-19.

Hence, it is important to understand if SARS-CoV-2 can infect and replicate in oral keratinocytes and fibroblasts, causing oral ulcerations and superficial necrosis before systemic symptoms [1,2].

To date, several studies have addressed the question of oral manifestations in patients positive with COVID-19 infection. Aragoneses et al., in their systematic review with a meta-analysis [2], highlight the relatively high frequency of xerostomia and aphthous lesions in patients with COVID-19.

Moreover, the presence of oral ulcers, vesicle-like herpes virus 1 (HSV1) infection, was also observed and Jimenez-Cauhe et al. detected erythema multiforme lesions as cutaneous and oral manifestations [3]. They concluded that Erythema Multiforme-like exanthem might be a peculiar pattern of exanthem associated with COVID-19.

Nonetheless, it remains unresolved if these oral health conditions are clinical consequences of COVID-19 infection or of faulty immune systems, resulting in the occurrence of potential co-infections with other viruses, bacteria or fungi, or adverse reactions to invasive drugs or treatments.

Erythema Multiforme (EM) is an acute, self-limited, immune-mediated condition characterized by the appearance of distinctive target-like lesions on the skin and mucosal involvement. A variety of factors were implicated in the pathogenesis of EM. It is considered to be a type IV hypersensitivity reaction associated with viral infections, medications and other various trigger factors. Herpes simplex virus and Mycoplasma pneumoniae are the main agents, causing skin manifestations [3].

Recent articles also report that SARS-CoV-2, in the same way as any other virus, can induce Type II and Type IV hypersensitivity reactions, in addition to a viral cytopathic effect. Specifically, it was illustrated that the severity of COVID-19 disease was linked to the high release of cytokines from host cells, referred to as a “cytokine storm” [4]. That evidence could explain the autoimmune component of SARS-CoV-2 infection and identify a relationship with EM.

Oral health conditions may be common in people with COVID-19 infection and should be considered in both the onset and progression of the disease. Clinicians in daily routine at the dental office should act as the primary prevention of oral diseases linked to SARS-CoV-2 infection. Knowing how to identify, and what they are looking for, is useful to not underestimate any anamnestic data or any lesions. This raised awareness could lead to an early diagnosis of COVID-19 infection, meaning early treatment of the possibly severe consequences.

This paper aims to increase awareness of EM lesions, as an early disease associated with COVID-19 infection, showing a case report of a young woman, who provided informed consent for publication.

## 2. Case Presentation

A 30 year-old Caucasian woman was referred to the Department of Oral and Maxillofacial Sciences of Sapienza University of Rome due to the presence of multiple hemorrhagic crusting and extensive erosions involving lips, ulcers on the hard palate, blisters and ulcers on the dorsal surface of the tongue and on cheek mucosa, which had appeared about 7 days ago. Before the specialist visit, her general practitioner prescribed antibiotic therapy as treatment. The reaction was massive, the lesions worsened and the patient had difficulty in speaking and eating as a result of the pain.

A medical history of the patient revealed no systemic pathologies or allergies. No history of HSV, or of taking medications in the last three months were reported by the patient, who, moreover, declared they had not received the COVID-19 vaccine.

**Figure 1:**
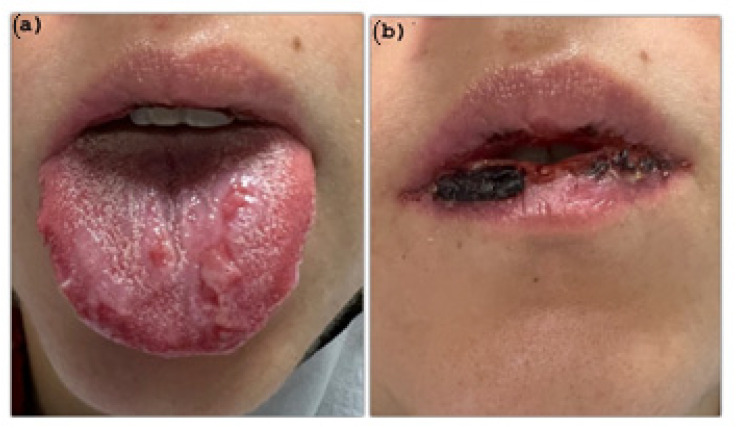
(**a**) blisters and ulcers on dorsal surface of the tongue; (**b**) necrotic tissue and hemorrhagic crusting on the vermilion lower lip.

**Figure 2:**
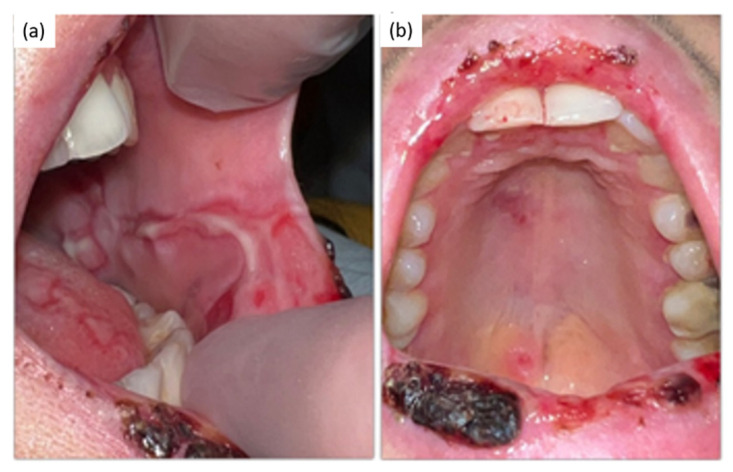
(**a**) blisters on the cheek mucosa; (**b**) ulcers on the palate.

Local examination of the mouth showed diffuse and symmetrical oral ulcerations aphthous-like in the buccal mucosa and a superficial necrosis with some hemorrhagic areas in the vermilion lips and in the posterior pharynx (Figure 1a,b and Figure 2a,b).

**Figure 3:**
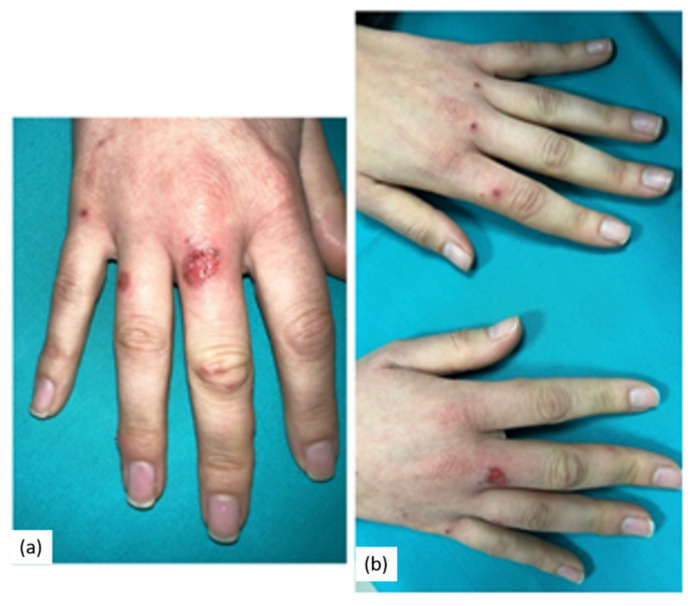
(**a**,**b**) cutaneous “target” lesions on the hands; since they have a typical poly cyclic or annular appearance with central erythematous-violet disc and a pink halo separated by a pale intermediate ring.

Bilateral cutaneous lesions were also evident on the hands. They consisted of itchy erythematous macules, with rounded, circulate erythema, known as target lesions (Figure 3a,b). No fever was observed.

### Diagnosis and Treatment

Based on the medical history and on the clinical evidence, the patient was diagnosed with Erythema Multiforme (EM) in its major form (Stevens–Johnson Syndrome (SJS)).

**Figure 4:**
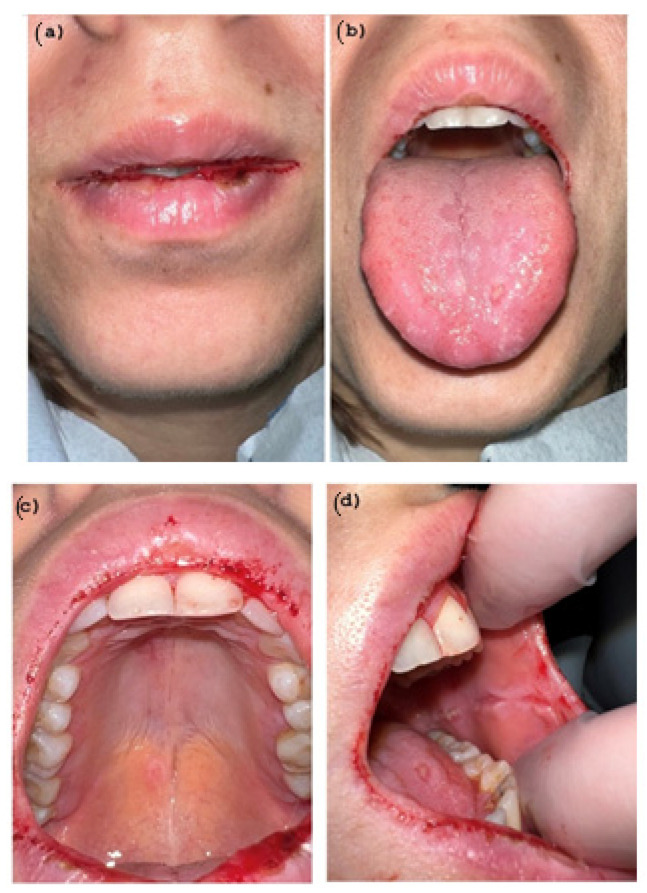

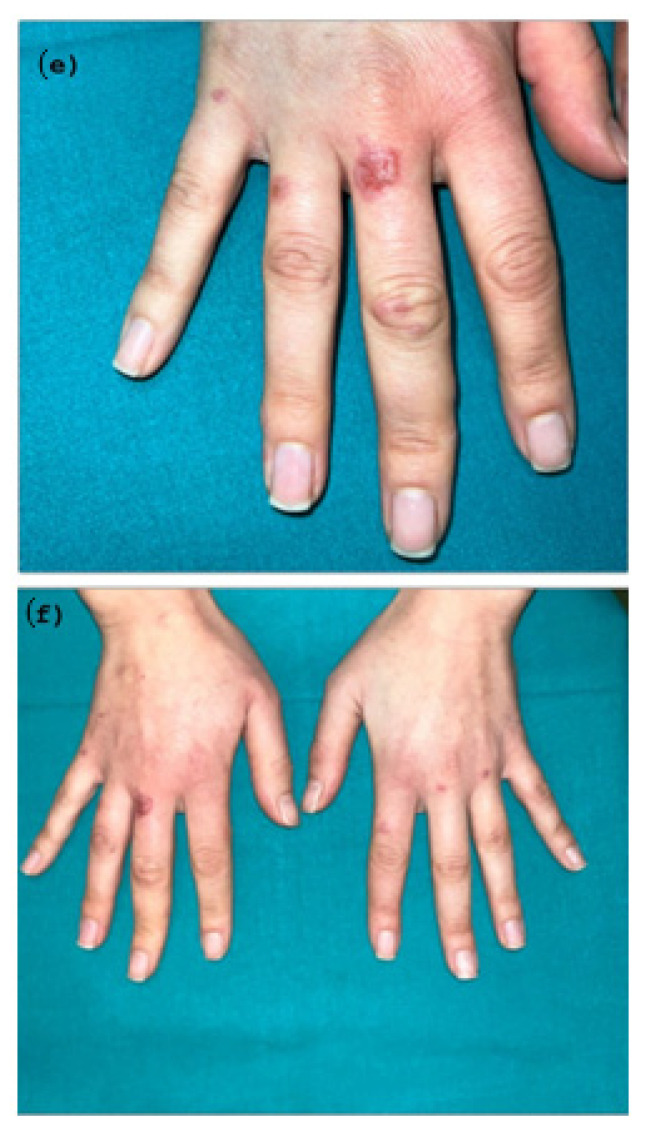
(**a**–**f**) Partial Healing wound after 5 days of Corticosteroid therapy. Hemorrhagic area and target lesions are still present.

The patient was prescribed Methylprednisolone 16 mg tablets once per day for 5 days, as well as mouthwashes with hyaluronic acid. A follow-up appointment was scheduled five days later; the lesions were in a stage of initial recovery (Figure 4a–f) but the patient started to have a low-grade fever, so a molecular nasopharyngeal test followed by a RT-PCR to detect COVID-19 was prescribed.

A second corticosteroid cycle was prescribed but the patient interrupted it because of the positivity of the COVID-19 test. The patient continued to take the hyaluronic acid mouthwashes.

A phone follow-up was arranged during the patient’s quarantine. She had fever and headache as the main symptoms of COVID-19.

**Figure 5:**
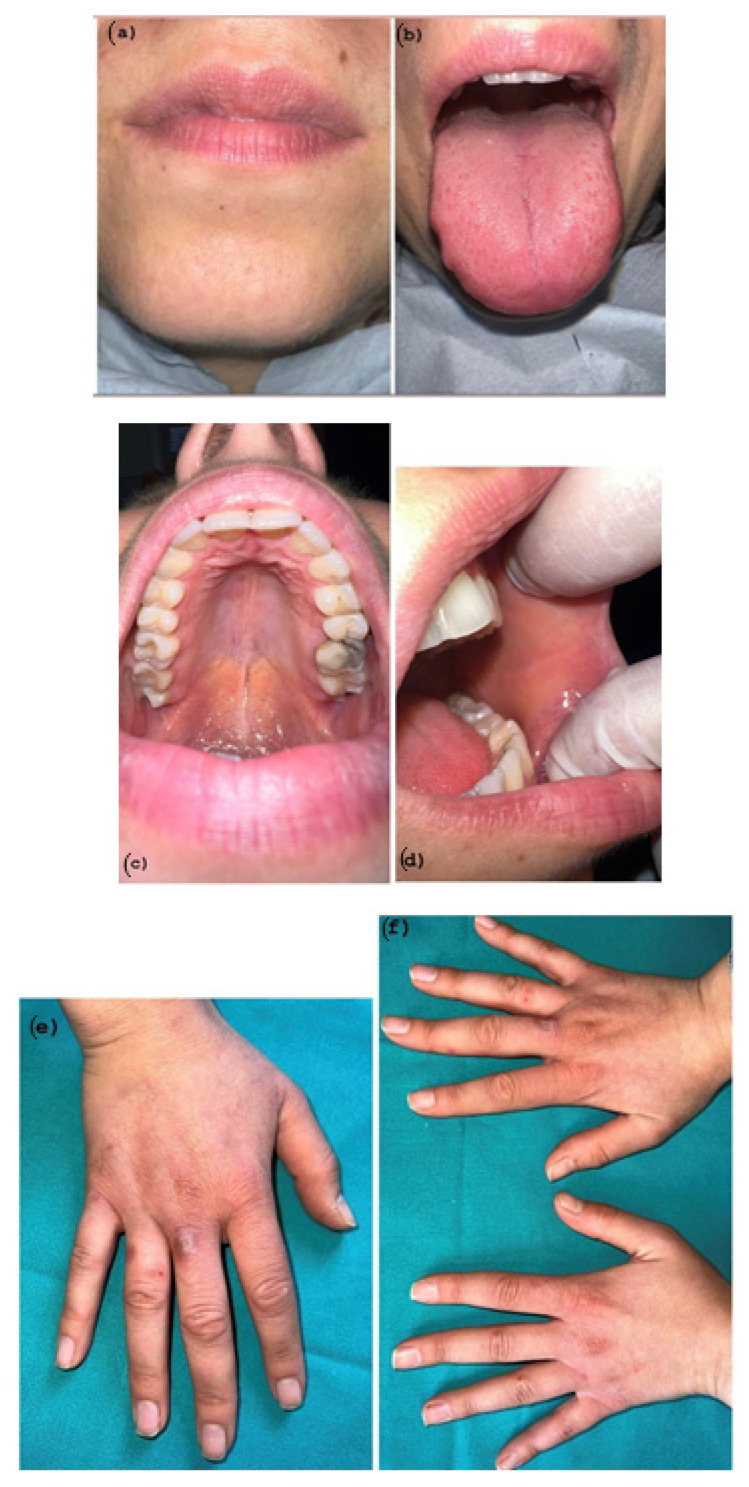
(**a**–**f**) Complete remission of the oral manifestations after 3 weeks. All the mucous and skin lesions regressed.

Complete remission of the oral manifestations was observed 3 weeks after initial admission to our Department (Figure 5).

Table 1 shows the temporality of the oral and skin manifestations and their relationship with the signs and symptoms of COVID-19 referred to. The intensity of pain was registered through the Visual Analogue Scale (VAS).

## 3. Discussion

In most people, the SARS-CoV-2 infection is mild and includes asymptomatic and pauci-symptomatic forms, and these persons successfully defend themselves from the virus via the triggering of innate immunity (Phase I). This is characterized by an early IFN response and then, over time, by induction of an adaptive immune response (Phase 2) with specific immunoglobulin production accompanied by T-cell activation and the generation of memory cells. For a minority of infected individuals, however, the disease is severe, especially for those with numerous comorbidities, who then may progress to pneumonitis, with a yet smaller percentage experiencing critical complications (acute respiratory failure, shock, immuno-thrombosis, multiorgan dysfunction or failure and death) (Phase 3). This phase is accompanied by an immune response characterized by hyperinflammation, reminiscent of a wide number of disorders, often collectively referred to as cytokine release or cytokine storm [5].

So, although the inflammation caused by SARS-CoV-2 infection is predominantly directed towards the respiratory system, some patients can develop an abnormal inflammatory reaction involving extrapulmonary tissues. Clinical backgrounds are very different and can resemble some autoimmune or inflammatory diseases, with signs and symptoms influenced by age, sex or ethnicity. Little is known about the pathogenesis of these manifestations [6].

Few studies have described the oral facial manifestations related to COVID-19 [1,2]. Dysgeusia and anosmia [7], even in the absence of respiratory symptoms, recognized in the early days of COVID-19 infection, represent the main signs and symptoms of this viral infection [7,8]. According to the review of Caposale et al., oral erosions, ulcers and blood crusts, gingival petechiae, macules, erythema multiforme and lesions frequently occur in COVID-19 patients [8].

Eghbali Zarch et al. assumed that such oral manifestations could be attributed to a co-existing immunosuppressive state that may lead to the risk of exacerbating existing weakened immune conditions via the cytokine storm [9,10]. Hence, the immune system plays a central role in the disease. The use of medicines, the nature of the virus and stress may negatively influence the immune system [8].

Such findings agree with the presented case report.

About the cutaneous manifestations in patients with COVID-19 infection, several syndromes were related to it [11,12]. Among these, EM lesions are of great importance [12,13]. EM is an acute, immune-mediated disease appearing with typical ‘target’ lesions [13]. The skin lesions consist of a polymorphous eruption of macules, papules and typical lesions that are symmetrically distributed in the distal extremities.

EM is linked to infective agents, and the Herpes Simplex virus and Mycoplasma pneumoniae are the main agents [13]. Therefore, EM-like eruptions, associated with COVID-19 infection, were classified into three patterns [14]: (1) the virus-related juvenile type, referring to patients aged less than 30 years; (2) the virus-related older type referring to patients older than 55 years; and (3) the drug-induced type. The presented case may belong to the first model.

To point out the diagnosis, Elboraey et al. described oral manifestations of Stevens–Johnson Syndrome (SJS) as polymorphic, erosive and erythematous lesions. This syndrome occurs mostly in adults between 20 and 40 years old, with similar manifestations to those reported in our case of multiple ulcerations, erythema and bullae [15]. Moreover, SJS is extremely rare with vaccination [15,16]; this is relevant information since the patient in this case had not received any vaccination against COVID-19.

In the presented case report, COVID-19 infection was detected, using a RT-PCR, about 10 days after the appearance of oral lesions. It cannot be ruled out that the infection was already present, as no swab was executed before the onset of the fever. WHO indicates that clinical symptoms could develop within 14 days of exposure, with a median incubation rate of approximately 5 to 7 days. Such considerations may suggest that the development of oral manifestations in this patient may be directly associated with the early COVID-19 expression (during the incubation period?). Whether the lesions were caused directly by the coronavirus or resulted from the severe compromised state of the patient that induces the viral infection remains to be determined.

## 4. Conclusions

The described case shows the first EM/SJS case as an earlier symptom of COVID-19 in an immune-competent young woman in the absence of any multi-organ symptoms.

Despite the limitations of the study, due to a single case reported and the difficulty in understanding the timing of the onset of febrile symptoms in the patient, the awareness and the need to not underestimate oral lesions and their plausible correlation with COVID-19 during daily activity at the dental office, as preventative action for avoiding the spread and the evolution of the disease, must be highlighted.

Further studies are needed to evaluate a new method for detecting early signs of COVID-19 and whether these lesions are associated with the virus, the drugs used or any other conditions [17].

## Figures and Tables

**Table 1:** Temporality of the oral and skin manifestations. The intensity of pain was registered through the Visual Analogue Scale (VAS).

Incubation Time	Oral and Cutaneous Lesions Occurence	Intensity of Pain (Vas)	Covid-19 Symptoms
Day 1	Oral vesicle in buccal and cheeck mucosa	4	None
Day 2	Oral vesicle in buccal and cheeck mucosa	5	None
Day 3	-Oral vesicle in buccal and cheeck mucosa -Blisters in the dorsum of the tongue	7	None
Day 4	-Oral vesicle in buccal and cheeck mucosa -Blisters in the dorsum of the tongue -Oral ulcerations- aphthous like in the vermillion lips	8	none
Day 5	-Oral vesicle in buccal and cheeck mucosa -Blisters in the dorsum of the tongue -Oral ulcerations- aphthous like in the vermillion lips -Target lesions in the hands	8	None
Day 6	-Oral vesicle in buccal and cheeck mucosa -Blisters in the dorsum of the tongue -Oral ulcerations- aphthous like in the vermillion lips -Oral ulcer in palate -Target lesions in the hands	8	None
Day 7	-Oral vesicle in buccal and cheeck mucosa -Blisters in the dorsum of the tongue -Oral ulcerations- aphthous like in the vermillion lips -Oral ulcer in palate -Target lesions in the hands	7	None
Day 8	-Oral vesicle in buccal and cheeck mucosa -Blisters in the dorsum of the tongue -Oral ulcerations- aphthous like in the vermillion lips -Oral ulcer in palate -Target lesions in the hands	6	None
Day 9	-Oral vesicle in buccal and cheeck mucosa -Blisters in the dorsum of the tongue -Oral ulcerations- aphthous like in the vermillion lips -Oral ulcer in palate -Target lesions in the hands	5	None
Day 10	-Oral vesicle in buccal and cheeck mucosa -Blisters in the dorsum of the tongue -Oral ulcerations- aphthous like in the vermillion lips -Oral ulcer in palate -Target lesions in the hands	3	None
Day 11	-Oral vesicle in buccal and cheeck mucosa -Blisters in the dorsum of the tongue -Oral ulcerations- aphthous like in the vermillion lips -Oral ulcer in palate -Target lesions in the hands	3	None
Day 12	-Hemorragic areas in the oral cavity -Target lesions (Partial Healing wound after 5 days of Corticosteroid therapy)	2	None
Day 13	-Hemorragic areas in the oral cavity -Target lesions Each lesions are in remission	2	Headache
Day 14	Each lesions are in remission	1	Fever and headache
